# Unveiling GATOR2 Function: Novel Insights from Drosophila Research

**DOI:** 10.3390/cells13211795

**Published:** 2024-10-30

**Authors:** Lucia Bettedi, Yingbiao Zhang, Shu Yang, Mary A. Lilly

**Affiliations:** 1 Eunice Kennedy Shriver National Institute of Child Health and Human Development, National Institutes of Health, Bethesda, MD 20892, USA; lucia.bettedi@edu.unife.it (L.B.); shu.yang2@nih.gov (S.Y.); 2 Institute for Translational Medicine, The Affiliated Hospital of Qingdao University, Qingdao 266000, China; zhangyingbiao@qdu.edu.cn

**Keywords:** Drosophila, GATOR2, GATOR1, TORC1, Wdr59, MIT/TFE, Rag GTPase, UNMET, species-specific nutrient sensor

## Abstract

The multiprotein Target of Rapamycin (TOR) Complex 1 (TORC1) is a serine/threonine kinase that stimulates anabolic metabolism and suppresses catabolism. Deregulation of TORC1 is implicated in various human pathologies, including cancer, epilepsy, and neurodegenerative disorders. The Gap Activity Towards Rags (GATOR) complex contains two subcomplexes: GATOR1, which inhibits TORC1 activity; and GATOR2, which counteracts GATOR1s function. Structural and biochemical studies have elucidated how GATOR1 regulates TORC1 activity by acting as a GTPase activating protein for Rag GTPase. However, while cryogenic electron microscopy has determined that the structure of the multi-protein GATOR2 complex is conserved from yeast to humans, how GATOR2 inhibits GATOR1 remains unclear. Here, we describe recent whole-animal studies in Drosophila that have yielded novel insights into GATOR2 function, including identifying a novel role for the GATOR2 subunit WDR59, redefining the core proteins sufficient for GATOR2 activity, and defining a TORC1-independent role for GATOR2 in the regulation of the lysosomal autophagic endomembrane system. Additionally, the recent characterization of a novel methionine receptor in Drosophila that acts through the GATOR2 complex suggests an attractive model for the evolution of species-specific nutrient sensors. Research on GATOR2 function in Drosophila highlights how whole-animal genetic models can be used to dissect intracellular signaling pathways to identify tissue-specific functions and functional redundancies that may be missed in studies confined to rapidly proliferating cell lines.

## 1. Introduction

TORC1 is a central regulator of metabolism in eukaryotes [1,2,3,4]. To maintain metabolic homeostasis, TORC1 integrates information from multiple signaling pathways that monitor a wide array of upstream inputs from both the intracellular and extracellular environments including amino acids, growth factors, ATP, and cholesterol levels [5]. In the presence of sufficient growth signals and nutrients, TORC1 phosphorylates downstream targets that promote translation, ribosome biogenesis, as well as other processes associated with anabolic metabolism and cell growth. Conversely, TORC1 actively inhibits catabolism and autophagy through the inhibitory phosphorylation of proteins that drive these processes including but not limited to ATG1/ULK1 and MiT/TFE transcription factors [6]. TORC1 deregulation contributes to many human pathologies including cancer, epilepsy, and aging. Thus, a basic understanding of the pathways that regulate TORC1 is the focus of intense interest.

**Figure 1:**
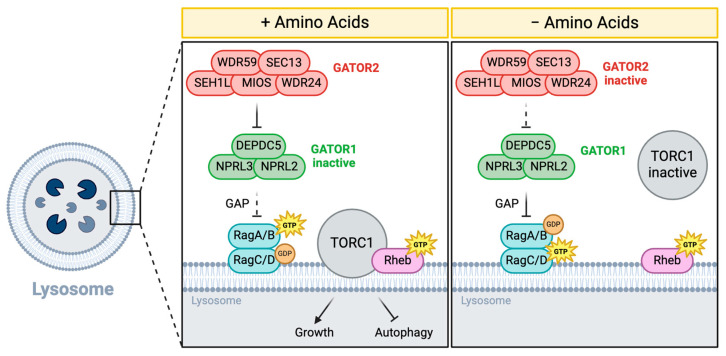
The GATOR complex regulates TORC1 activity in response to amino acid availability. The GATOR complex is an upstream regulator of TORC1 that is composed of two multiprotein sub-complexes, GATOR1 and GATOR2. The GATOR1 complex inhibits TORC1 activity in response to amino acid starvation by acting as a GTPase-activating protein towards the RagA/B subunit of Rag GTPase. Rag GTPase recruits TORC1 to lysosomes for activation by Rheb. GATOR2 opposes the activity of GATOR1 and thus functions to activate TORC1.

The activity of TORC1 is regulated by changes in its intracellular localization [7]. In the presence of sufficient growth signals and nutrients, TORC1 is recruited to lysosomes by Rag GTPase, where it is activated by small GTPase Rheb [8,9,10,11] (Figure 1). Rag GTPase is an obligate heterodimer composed of two small GTPases, a RagA/B subunit bound to a Rag C/D subunit, and is anchored to the lysosomal membrane through the scaffold protein complex Ragulator [7,12,13]. In the presence of amino acids, the active form of RagA/B (GTP-loaded) recruits TORC1 to lysosomes, facilitating its activation by Rheb. Rag GTPases are modulated by the **G**ap **A**ctivity **TO**ward **R**ags (**GATOR**) complex [14,15], which was first identified in yeast and named the **Se**h1 **A**ssociate (**SEA**) complex [16] (Table 1). In this review, we primarily use the mammalian nomenclature. Evidence from yeast and mammals indicates that the three components of the GATOR1 complex, DEPDC5, NPRL2, and NPRL3, function as GTPase-activating proteins (GAPs) that inhibit TORC1 activity by promoting the hydrolysis of RagA^GTP^ to its inactive RagA^GDP^ state [14,15]. Consistent with reports for yeast and mammalian cultured cells, whole-animal studies in Drosophila confirm that the GATOR1 complex is required for the maintenance of metabolic homeostasis and the response to nutrient stress at both the cellular and organismal levels [17,18,19,20].

The GATOR2 complex inhibits GATOR1 and thus functions to promote TORC1 activity [14,18,21]. GATOR2 is a large (1.1 MDa) multiprotein complex comprising the conserved proteins WDR24, MIOS, WDR59, SEH1L, and SEC13. Three of the proteins, WDR24, MIOS, and WDR59, contain RING domains, which are required for both the integrity and the function of the complex, as well as N-terminal β-propellers composed of WD40 repeats [22,23,24]. Early computational analysis from yeast showed that GATOR2 is predicted to contain structural features characteristic of coatomer proteins and membrane tethering complexes [25,26]. Consistent with the structural and computational analysis, GATOR2 localizes to the surface of the lysosomes and autolysosomes in Drosophila and mammalian tissues and to the vacuolar membrane in yeast [14,18,20,24,25,27]. Recent cryo-EM studies revealed the GATOR2 complex, known as SEACAT in yeast, adopts a large, two-fold symmetric, cage-like structure composed of two WDR24-SEH1L, four MIOS-SEH1, and two WDR59-SEC13 heterodimers, which form an octagonal scaffold supporting eight pairs of WD40 β-propellers [23,24]. While the detailed structure of the GATOR2 complex is conserved from yeast to humans, precisely how GATOR2 inhibits GATOR1 remains unknown.

### WDR59 Is an Upstream Inhibitor of GATOR2

In the initial functional characterization of GATOR2 in cultured mammalian and *Drosophila* cells, WDR59 was found to be required for the GATOR2-driven inhibition of GATOR1 [14]. Specifically, RNAi knockdowns of the GATOR2 components MIOS, SEH1L, WDR24, and WDR59 decrease TORC1 activity in Drosophila S2 cells and mammalian HEK-293T cells [14,22,23,28]. Furthermore, CRISPR/Cas9-mediated knockouts of WDR59 in various cancer cell lines, including HeLa and SUM159, resulted in reduced TORC1 signaling and growth inhibition [23,28,29,30,31]. These findings support the conclusion that WDR59 works in concert with other GATOR2 components to suppress GATOR1 activity, thereby facilitating the RagA GTPase-mediated recruitment of TORC1 to the lysosomes for activation by Rheb (Figure 1).

However, functional analysis of both single-cell and multicellular eukaryotes indicates that WDR59, known as SEA3 in yeast, plays a unique role in regulating the GATOR-Rag GTPase-TORC1 signaling axis. While deleting WDR59 in cultured cells leads to reduced TORC1 activity, similar to deletions of other GATOR2 subunits, WDR59 deletions in HEK-293T cells and mouse embryonic fibroblasts cause partial resistance to nutrient deprivation, a phenotype that suggests reduced GATOR1 functionality [29]. Similarly, in the yeast *Schizosaccharomyces pombe* (*S. pombe*), SEA3 knockouts partially phenocopied knockouts of the GATOR1 complex components IML1, NPR2, and NPR*3*, showing increased TORC1 activity and reduced response to nitrogen limitation [21,32]. Cryo-EM analysis from both yeast and humans indicates that WDR59/SEA3 impacts the structure of both the GATOR2 and GATOR1 complexes [23,24]. Specifically, the C-terminus of the WDR59/SEA3 protein contributes to the GATOR2 core complex, while the N-terminus of the protein mediates an interaction with GATOR1.

**Figure 2:**
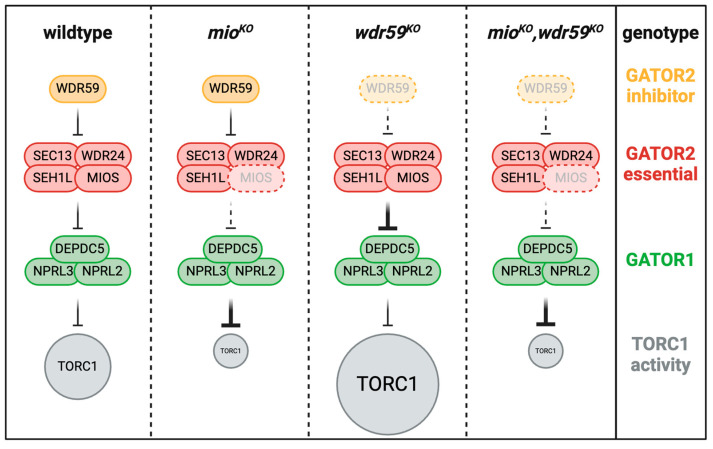
Wdr59 is an upstream inhibitor of GATOR2 in Drosophila. In wildtype animals, Wdr59 inhibits TORC1 activity upstream of the GATOR2 complex. In *mio^KO^* mutants, the GATOR1 complex is constitutively active in the female germline, resulting in reduced TORC1 activity. In *wdr59^KO^* mutants, GATOR2 increases its association with GATOR1, further inhibiting GATOR1 activity and allowing for the increased activation of TORC1. Double mutants of *wdr59^KO^*/*mio^KO^* phenocopy the *mio^KO^* single mutants. Thus, Wdr59 requires a functional GATOR2 complex to inhibit TORC1 activity.

How might WDR59 promote the activity of both GATOR1 and the GATOR1 inhibitor, GATOR2? Recent work from *Drosophila* coupled with the structural analysis suggests a potential answer to this question [27]. In *Drosophila*, females containing individual knockouts of the GATOR2 components MIO, SEH1, and WDR24 have small ovaries with reduced TORC1 activity [18,20,33,34]. These ovarian phenotypes are reversed in GATOR1/GATOR2 double mutants, consistent with the standard model that the primary function of the GATOR2 complex is to restrain the activity of the TORC1 inhibitor, GATOR1 [18,20] (Figure 2). However, unlike the other GATOR2 mutants, WDR59 knockout (*wdr59^KO^)* mutant females have large ovaries and are fully fertile [27]. Furthermore, ovaries from *wdr59^KO^* females have increased levels of TORC1 activity relative to wildtype ovaries. Consistent with these genetic studies, biochemical analysis showed that in *wdr59^KO^* ovaries, the association of GATOR1 with Rag GTPase decreased, consistent with the decreased ability of the GATOR1 complex to inhibit Rag GTPase [27]. Thus, in the Drosophila ovary, like GATOR1, WDR59 functions to inhibit TORC1 activity.

However, does WDR59 function as an obligate component of the GATOR1 in Drosophila, as is the case with the SEA3 homolog in *S. pombe*? Researchers performed epistasis analysis to address this question [27]. If WDR59 is a component of GATOR1, it should act independently of its upstream inhibitor, GATOR2 (Figure 1). In other words, the WDR59 phenotype should be unimpacted by reduced GATOR2 activity. Double-mutant analysis in Drosophila found the opposite is true. While *wdr59^KO^* single mutants have full-sized ovaries and high TORC1 activity, WDR59/GATOR2 double mutants (*wdr59^KO^, wdr24^KO^*, and *wdr59^KO^, mio^KO^*) have small ovaries with diminished TORC1 activity, similar to the phenotypes observed in the GATOR2 single mutant (Figure 2). Thus, in the Drosophila ovary, contrary to observations in *S. pombe,* the TORC1 inhibitory function of WDR59 requires the presence of a functional GATOR2 complex [27]. These data strongly suggest that in Drosophila, WDR59 is not a component of GATOR1 but instead functions as an upstream inhibitor of GATOR2. Additionally, in *wdr59^KO^* ovaries, the interaction between GATOR1 and GATOR2 was elevated, while the interaction between GATOR1 and Rag GTPase was reduced. This decrease aligns with the diminished capacity of the GATOR1 complex to inhibit Rag GTPase. The most parsimonious model explaining these data is that the absence of WDR59, GATOR2 strongly inhibits GATOR1 function, phenocopying GATOR1 mutants. Notably, this phenotype partially overlaps with that of SESTRIN mutants in Drosophila [35,36,37]. SESTRIN2 is a well-characterized upstream inhibitor of GATOR2 that functions, at least in part, as an amino acid sensor, connecting amino acid availability to TORC1 activity [35,38,39].

This raises the question of why WDR59 is necessary for GATOR2 function in mammalian cells but inhibits GATOR2 in numerous Drosophila cell types [14,27]. In mammalian cells, the multiprotein KICSTOR complex is essential for localizing GATOR1 on lysosomal membranes and for maintaining GATOR1 protein stability [29,40]. KICSTOR is not conserved in Drosophila. Could similar differences exist in the proteins that support GATOR2 function between Drosophila and mammals? Notably, in WDR59 knockout HeLa cells, there is a significant reduction in the levels of the GATOR2 components MIOS and WDR24. Additionally, treating WDR59 knockout HeLa cells with a proteasome inhibitor markedly increased the levels of GATOR2 proteins and partially restored TORC1 activity [27]. In contrast, in Drosophila ovarian and imaginal disc tissues, the levels of MIO and WDR24 are not impacted by the presence or absence of the WDR59 subunit [27]. One plausible explanation for these findings is that in cultured mammalian cells, WDR59 is required for GATOR2 function because it protects the GATOR2 complex from proteasomal degradation. Surprisingly, this role for WDR59 may not be restricted to mammals. In the Drosophila fat body, unlike what is observed in ovarian and imaginal disc tissues, WDR59 promotes TORC1 activity as well as the accumulation of GATOR2 complex components MIO and SEH1 [27]. Similarly, WDR59 promotes TORC1 activity in Drosophila S2 cells [14]. Taken together, these data support a model in which the role of WDR59 in regulating TORC1 activity depends on the cellular and/or metabolic context [27]. Therefore, an important avenue for future research involves elucidating the molecular mechanisms through which WDR59 regulates the stability and activity of GATOR2.

## 2. GATOR2 Regulates Lysosome Function Independent of TORC1 Activity

The lysosomal–autophagic system regulates the balance between anabolism and catabolism, which is essential to metabolic homeostasis [41,42,43]. During periods of nutrient stress, cells upregulate lysosomal function and the autophagic process to promote the catabolic breakdown of macromolecules such as proteins and lipids to provide building blocks for cells to reuse. The MiT/TFE family promotes lysosomal–autophagic function by stimulating the transcription of numerous genes related to the lysosomal–autophagic system, including lysosomal lumen enzymes, V-ATPase, and ATG proteins [44]. A large body of work from multiple laboratories has provided a detailed mechanism for how TORC1 functions to inhibit the activation of MiT/TFE proteins through multiple mechanisms, including cytoplasmic retention and proteolytic degradation [45,46,47,48,49,50,51]. These regulatory pathways allow MiT/TFEs to transcribe the genes required to promote catabolic metabolism during periods of nutrient stress when TORC1 activity is low.

Thus, it was surprising when it was reported that GATOR2, known as an activator of TORC1, is essential for lysosomal function [28]. Research in Drosophila first demonstrated that mutations in genes encoding the GATOR2 subunits, WDR24, MIO, and SEH1, result in the accumulation of abnormal autolysosomes and defects in autophagic influx [20]. Interestingly, restoring TORC1 activity by creating GATOR2/GATOR1 double mutants did not rescue the abnormal lysosome phenotypes. These data support the novel conclusion that GATOR2 has a second critical target that promotes lysosome function independent of GATOR1 and the Rag GTPAse-TORC1 signaling pathway [20].

**Figure 3:**
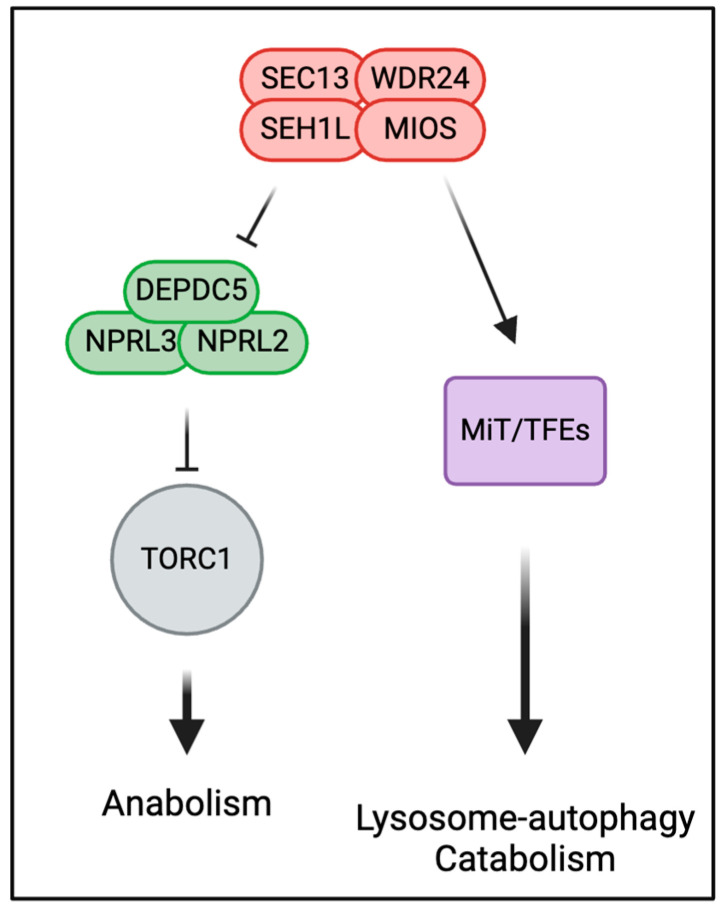
GATOR2 has two independent functions. First, GATOR2 inhibits GATOR1 to promote TORC1 activity. Second, in a pathway that is independent of the Rag GTPase and TORC1 activity, GATOR2 promotes lysosomal autophagic function by ensuring the activity of MiT/TFE transcription factors.

As an extension of this work in Drosophila, subsequent studies with mammalian cell cultures determined that the GATOR2 subunits WDR24 and MIOS maintain lysosomal function by preventing the proteasomal degradation of MiT/TFE transcription factors [28]. Knockouts of WDR24 and MIOS significantly reduced the protein levels of MiT/TFE family members TFEB, TFE3, and MITF, resulting in the decreased expression of lysosomal genes and the reduced ability of lysosomes to digest cargo [28]. Further analysis revealed that the low levels of MiT/TFE proteins in GATOR2 knockout cells is due to increased proteasomal degradation. Importantly, epistasis analysis showed that the requirement for GATOR2 to protect the stability of MiT/TFE proteins is independent of both GATOR1 and Rag GTPase. Thus, in the absence of GATOR2, cells have both reduced TORC1 and MiT/TFE activities. Returning to the Drosophila genetic model, GATOR2 was found to promote the accumulation of MITF protein as well as the expression of an array of lysosomal autophagic genes (Figure 3). Taken together, these data indicate that GATOR2 has a second evolutionarily conserved function, which is independent of the GATOR1-Rag-GTPase-TORC1 signaling pathway, in the regulation of the lysosomal-autophagic membrane system.

The hypothesis that GATOR2 targets two independent pathways, TORC1 activation and lysosomal function, is supported by work on the GATOR2 component WDR59. Notably, in both cultured Drosophila and mammalian cells, lysosomal function appears unimpacted in WDR59 knockout cells [27,28]. Moreover, while the levels of MiT/TFEs are dramatically reduced in WDR24 knockout HeLa cells, this is not the case in WDR59 knockout cells, which exhibit levels of TFE3 and MITF proteins similar to those of controls, with only a modest reduction in the level of TFEB [28]. These data are consistent with the model that WDR59 acts upstream of the GATOR2 complex to mediate the association of GATOR1 and the Rag GTPase. Moreover, they support the idea that the core GATOR2 complex targets two independent pathways: one that controls TORC1 signaling through the inhibition of GATOR1 and a second required to maintain the stability of MiT/TFE transcription factors and the expression of lysosomal autophagic genes (Figure 3).

## 3. GATOR2 and a Model for the Evolution of Species-Specific Nutrient Sensors

Recent work in Drosophila describes how the GATOR2 complex serves as a platform to integrate a species-specific metabolic sensor into the TORC1 signaling pathway [52]. Liu, Sabatini, Perrimon, and colleagues reasoned that because species have distinct dietary needs and environmental niches, they have likely evolved novel independent mechanisms to sense specific nutrients. In mammals, multiple nutrient sensors interact with components of the GATOR2 complex, including SESTRIN2, a leucine sensor that binds WDR24, and CASTOR1, an arginine sensor that associates with MIOS [35,36,38,40,53]. While Drosophila contains a SESTRIN2 homolog, it does not contain an obvious homolog for CASTOR1.

To identify novel nutrient sensors, researchers searched for proteins that are associated with the GATOR2 complex in Drosophila but not in mammalian cells. From these experiments, they identified the UNMET protein, which is conserved from yeast to humans [52]. Work in multiple other species indicates that UNMET acts as a carnosine N-methyltransferase, although whether it has retained this enzymatic activity in Drosophila is not known [54]. Perhaps more intriguingly, earlier reports from humans indicate that UNMET binds the methionine-derived methyl donor S-adenosylmethionine (SAM) [55]. Notably, in mammals, the levels of the metabolite SAM are sensed by the protein SAMTOR1, which feeds directly into the response to methionine levels via the GATOR1-KICSTOR complex [56].

Might UNMET function as a species-specific methionine sensor in Drosophila? Consistent with this idea, depletions of UNMET made the TORC1 pathway in Drosophila S2R+ cells unresponsive to changes in methionine levels while leaving sensitivity to leucine, threonine, glutamine, phenylalanine, and tryptophan unaffected. Moreover, the overexpression of UNMET inhibited TORC1 activity. Finally, whole-animal studies revealed that ovaries from *unmet^KO^* mutant females fed a diet low in methionine contained an increased rate of apoptosis in early egg chambers relative to controls. This phenotype is similar to that previously reported in the ovaries of GATOR1 mutants, which retain inappropriately high TORC1 activity under conditions of amino acid starvation [17,19]. In sum, in Drosophila, UNMET is essential to regulating TORC1 activity in response to methionine restriction [52].

Next, the researchers examined if UNMET, as is observed with the SAMTOR1 protein in humans, indirectly monitors methionine levels through its ability to bind to the methionine metabolite SAM [52]. They found that SAM robustly disrupted the association of UNMET with GATOR2 in lysates containing the UNMET-GATOR2 complex derived from amino-acid-starved cells. Moreover, in cultured cells, expressing a mutated UNMET protein that cannot bind SAM results in the constitutive association of GATOR2 with UNMET and low TORC1 activity. Taken together, these data support the model that Drosophila responds to methionine levels through the SAM-dependent inhibition of the GATOR2 complex by UNMET. Thus, both humans and Drosophila monitor SAM levels as a proxy for methionine to link methionine availability to TORC1 activity. The evolution of UNMET in flies and SAMTOR1 in mammals appears to have occurred independently, indicating convergent evolution towards sensing the same metabolite, SAM.

Phylogenetics analysis combined with structure–function studies revealed that it is the MIO subunit of the GATOR2 complex that has evolved to bind UNMET in Drosophila. GATOR2 acquired the ability to bind UNMET late in insect evolution, at the appearance of the Order Diptera. The diverged amino acids in MIO between humans and Drosophila are on the outer exposed flexible loops of the protein and are not predicted to impact the ability of the MIO protein to generate the structural folds required for the formation of the core GATOR2 complex structure. The authors speculate that this may represent just one example of how nutrient sensors may evolve by utilizing pre-existing proteins that bind nutrient-relevant metabolites and can be integrated into a conserved nutrient signaling pathway [52].

## 4. Conclusions

Whole-animal studies in Drosophila have broadened our understanding of the GATOR-TORC1 signaling axis in metazoans and highlight the complexity of metabolic regulation in vivo. Because of its role in cancer, epilepsy, and neurodegeneration, the GATOR-TORC1 signaling pathway provides a target for pharmaceutical intervention [5]. Essential to this line of investigation is a full mechanistic understanding of the in vivo function of the GATOR2 complex in the regulation of TORC1 signaling, growth, and other cellular processes. Early studies conducted in Drosophila identified specific requirements for individual GATOR2 subunits across different tissues, insights not previously uncovered in cultured cells [18,20,27,33,34]. Subsequently, studies in mammals have identified tissue-specific requirements for several GATOR2 components [57,58]. Over the last several years, work in Drosophila has revealed novel mechanistic insights into the basic function of the GATOR2 complex, including identifying WDR59 as an upstream inhibitor of GATOR2 and demonstrating that GATOR2 has critical TORC1-independent functions in the regulation of the lysosomal–autophagic system. Finally, the recent identification of a species-specific methionine sensor in Drosophila has led to a new way of thinking about how nutrient sensors might have evolved [52]. In summary, Drosophila provides an excellent model for the molecular genetic dissection of the GATOR2 complex and its regulation of metabolic homeostasis at both the cellular and organismal levels.

## Figures and Tables

**Table 1:** The GATOR complex is conserved from yeast to humans. Homologs of GATOR2 (red) and GATOR1 (green) components in yeast, fruit fly, and human. In Drosophila Wdr59 (orange) functions as an inhibitor of GATOR2 activity.

*Saccharomyces cerevisiae*	*Drosophila melanogaster*	*Homo sapiens*
Sea3	Wdr59	WDR59
Sec13	Sec13	SEC13
Sea2	Wdr24	WDR24
Seh1	Seh1	SEH1L
Sea4	Mio	MIOS
Sea1	Iml1	DEPDC5
Npr2	Nprl2	NPRL2
Npr3	Nprl3	NPRL3
